# Long-Term Exposure to PFE-360 in the AAV-α-Synuclein Rat Model: Findings and Implications

**DOI:** 10.1523/ENEURO.0453-18.2019

**Published:** 2019-12-17

**Authors:** Michael Aagaard Andersen, Florence Sotty, Poul Henning Jensen, Lassina Badolo, Ross Jeggo, Garrick Paul Smith, Kenneth Vielsted Christensen

**Affiliations:** 1 Neurodegeneration, Neuroscience Drug Discovery DK, H. Lundbeck A/S, DK-2500 Valby Denmark; 2 Department of Biomedicine, Dandrite, Faculty of Health, Aarhus University, DK-8000 Aarhus Denmark; 3 Department of Discovery DMPK, H. Lundbeck A/S, DK-2500 Valby Denmark; 4 Department of Discovery Chemistry 2, H. Lundbeck A/S, DK-2500 Valby Denmark

**Keywords:** AAV-α-synuclein, α-synuclein, LRRK2, Parkinson’s disease, PFE-360, subthalamic nucleus

## Abstract

Parkinson’s disease (PD) is a progressive neurodegenerative disorder associated with impaired motor function and several non-motor symptoms, with no available disease modifying treatment. Intracellular accumulation of pathological α-synuclein inclusions is a hallmark of idiopathic PD, whereas, dominant mutations in leucine-rich repeat kinase 2 (LRRK2) are associated with familial PD that is clinically indistinguishable from idiopathic PD. Recent evidence supports the hypothesis that an increase in LRRK2 kinase activity is associated with the development of not only familial LRRK2 PD, but also idiopathic PD. Previous reports have shown preclinical effects of LRRK2 modulation on α-synuclein-induced neuropathology. Increased subthalamic nucleus (STN) burst firing in preclinical neurotoxin models and PD patients is hypothesized to be causally involved in the development of the motor deficit in PD. To study a potential pathophysiological relationship between α-synuclein pathology and LRRK2 kinase activity in PD, we investigated the effect of chronic LRRK2 inhibition in an AAV-α-synuclein overexpression rat model. In this study, we report that chronic LRRK2 inhibition using PFE-360 only induced a marginal effect on motor function. In addition, the aberrant STN burst firing and associated neurodegenerative processes induced by α-synuclein overexpression model remained unaffected by chronic LRRK2 inhibition. Our findings do not strongly support LRRK2 inhibition for the treatment of PD. Therefore, the reported beneficial effects of LRRK2 inhibition in similar α-synuclein overexpression rodent models must be considered with prudence and additional studies are warranted in alternative α-synuclein-based models.

## Abstract (precis)

Parkinson’s disease (PD) is a progressive neurodegenerative disorder associated with impaired motor function and several non-motor symptoms, with no available disease modifying treatment. Intracellular accumulation of pathological α-synuclein inclusions is a hallmark of idiopathic PD, whereas, dominant mutations in leucine-rich repeat kinase 2 (LRRK2) are associated with familial PD that is clinically indistinguishable from idiopathic PD.

## Significance Statement

Mutations in leucine-rich repeat kinase 2 (LRRK2) and α-synuclein are known risk factors for Parkinson’s disease (PD). α-Synuclein aggregates at autopsies in both idiopathic and most G2019S cases is suggestive of a common disease pathogenesis. LRRK2 and α-synuclein interaction is hypothesized to play a pivotal role in the pathologic mechanisms. Preclinical *in vivo* evidence of a beneficial effect of LRRK2 inhibition is mixed and limited. This study increases our understanding but underlines the complexity of LRRK2 as a mediator of neuronal dysfunction; importantly, the present findings further outline some of the limitations inherent to the PD model used, and warrant additional preclinical studies in animal models with better relevance to the clinical pathophysiology to draw conclusion on the therapeutic potential of LRRK2 modulation in PD.

## Introduction

Historically, neurodegeneration of dopaminergic (DAergic) neurons in substantia nigra pars compacta (SNc) and α-synuclein inclusions in Lewy bodies and Lewy neurites are the histopathological hallmarks of Parkinson’s disease (PD; Spillantini et al., 1997; Antony et al., 2013). According to the hypothesis raised by Braak and Beach, pathogenic α-synuclein species may be capable of transmitting their pathologic properties across brain nuclei following neuronal/axonal pathways (Braak et al., 2003, 2004; Beach et al., 2009). A number of known dominant autosomal missense mutations, duplications and triplications in the SNCA gene encoding α-synuclein are risk factors for developing PD (Pankratz et al., 2007; Lill, 2016). The exact physiologic role of α-synuclein is not fully understood. However, virally and genetically manipulated animal models have revealed impairments in synaptic transmission and vesicular machinery (Cabin et al., 2002; Yavich et al., 2004; Watson et al., 2009; Nemani et al., 2010; Busch et al., 2014; Subramaniam et al., 2014), suggesting an involvement of α-synuclein in normal synaptic neurotransmission.

Increased leucine-rich repeat kinase 2 (LRRK2) kinase activity is suggested as the key risk factor associated with late onset PD (Funayama et al., 2002; Paisán-Ruíz et al., 2004; Zimprich et al., 2004a,b; Di Fonzo et al., 2005, 2006; Gilks et al., 2005; Kachergus et al., 2005; Nichols et al., 2005; Healy et al., 2008; Ross et al., 2011). The physiologic function of LRRK2 kinase has recently been assigned as a controller of RAB GTPase activity via increased phosphorylation of several small RAB GTPase, and autophosphorylation (LRRK2-pS1292) of disease relevant mutant LRRK2 (Steger et al., 2016, 2017; Fan et al., 2017; Liu et al., 2017; Thirstrup et al., 2017). Genetic ablation and pharmacological inhibition of LRRK2 have previously been demonstrated to have a promising effect on disease relevant alterations in preclinical *in vivo* models of PD (Lin et al., 2009; Daher et al., 2014, 2015; Andersen et al., 2018a). The partly shared clinical and histopathological manifestation of idiopathic/sporadic and LRRK2 PD is suggestive of at least some shared physiopathological mechanisms between LRRK2 PD and sporadic PD, thereby supporting a potential for LRRK2 inhibition in the modulation of common pathways in the disease mechanisms.

Glutamatergic neurons in the subthalamic nucleus (STN) are a key regulator of neuronal input to the motor thalamus (Nambu et al., 2002). In PD patients and neurotoxin as well as viral models of PD, the loss of striatal DAergic neurotransmission triggers an increase in STN burst discharge pattern, and aberrant oscillations in the beta range throughout the basal ganglia (Brown, 2007; Steigerwald et al., 2008; Wilson et al., 2011; McConnell et al., 2012; Pan et al., 2016; Andersen et al., 2018a). Functional pre-clinical studies and deep brain stimulation studies in both PD models and humans suggest that functional relief of aberrant STN burst firing is associated with acute normalization of motor function (Grill et al., 2004; Benabid et al., 2009; McConnell et al., 2012; Moran et al., 2012; Pan et al., 2016; Wichmann and DeLong, 2016). Recently, acute effects of LRRK2 inhibition have been reported on α-synuclein-induced aberrant STN burst firing (Andersen et al., 2018a), possibly representing a disease relevant measurement to evaluate new disease modulatory targets.

In the present study, AAV-α-synuclein [human wild type (hwt)] overexpression in rat substantia nigra recapitulates PD-like aberrant STN burst firing, DAergic neurodegeneration and motor dysfunction. Using the AAV-α-synuclein overexpression approach, we investigated the effect of chronic LRRK2 inhibition on α-synuclein-induced neuronal dysfunction. Chronic LRRK2 inhibition only had a marginal effect on motor function, which was not associated with any effect on aberrant STN firing, DAergic neurodegeneration measured by striatal tyrosine hydroxylase expression, LRRK2 expression, α-synuclein expression or phosphorylation (α-synuclein-pS129). The present findings do not strongly support the mechanistic link between LRRK2 kinase activity and pathologic α-synuclein, and further reveal some limitations of the preclinical AAV α-synuclein overexpression model.

## Materials and Methods

### *In vivo* experimental methods

#### Ethical statement

All experiments were conducted in accordance with the European Communities Council Directive (86/609/EEC) for the care and use of laboratory animals and the Danish legislation regulating animal experiments.

### Animals

Female Sprague Dawley rats were acquired from Taconic Denmark (NTac:SD, *n* = 23 in total). Rats were group housed in humidity (55–65%) and temperature (22 ± 1.5°C) controlled conditions in a 12/12 h light/dark cycle. Water and food were available *ad libitum.* Female rats were used as experimental subject as they have lower body weight and lower variation in skull size compared to male rats at 26 weeks of age where the experiment was conducted. Lower body weight and variation in skull size are preferred in the *in vivo* electrophysiological experiments.

### Viral-mediated α-synuclein overexpression in substantia nigra

Injection of recombinant adeno-associated-viral vectors 2/5 (AAV) was performed in 10- to 12-week-old animals (weighing 250–300 g). Surgical neuroleptic analgesia was induced with a mixture of Hypnorm and Dormicum (corresponding to 157 μg/kg fentanyl, 2.5 mg/kg midazolam and 5 mg/kg fluanisone). All incisions were infiltrated with local analgesia (lidocaine). The rats were placed in a stereotaxic frame and the body temperature was maintained at 37.5 °C. After a skin incision, a hole was drilled in os paritalis directly above the SNc at the following coordinates, according to Paxinos and Watson (2007): 5.5 mm caudal to bregma and 2.0 mm lateral to the midline. The injection cannula (Hamilton, 30-gauge stainless-steel cannula) was slowly lowered into the injection site located 7.2 mm ventral to the brain surface. A unilateral injection of 3-μl AAV viral vector (3 × 10^10^ GC/ml; Vector Biolabs) containing the transgene for hwt α-synuclein or an empty vector (CTRL) was performed at a flow rate of 0.2 μl/min. The cannula was left in place for 5 min after the injection to allow diffusion. After the skin was sutured, a 24-h analgesic treatment protocol was initiated (buprenorphine 0.05 mg/kg every eight hour). The transgene expression was driven by a chimeric promoter element containing part of the cytomegalovirus promoter and a part of the synthetic chicken β-actin promoter. Further, the expression was enhanced by the woodchuck of hepatitis virus post-translational regulatory element. A successful viral injection was further validated by detection of hwt α-synuclein in the ipsilateral striatum by SDS-PAGE and subsequent Western blotting.

### PFE-360 treatment

PFE-360 (Baptista et al., 2015) was synthesized at H. Lundbeck A/S as described in patent application US2014/0005183 (example 217; 1-methyl-4-(4-morpholino-7H-pyrrolo[2,3-d] pyrimidin-5yl) pyrrole-2-carbonitrile). PFE-360 was dissolved at a concentration of 3 mg/ml in a vehicle containing 10% captisol and adjusted to pH 2 with 1 M methane sulfonic acid. The solution was administrated by oral gavage using plastic feeding tubes. Rats receiving PFE-360 were dosed with 7.5 mg/kg twice daily with a 12-h interval. The treatment was initiated on day 1 after AAV injection. The dose of 7.5 mg/kg was chosen based on a separated pharmacokinetic study showing that 7.5 mg/kg gives full LRRK2 kinase inhibition from 1 to 10 h and ∼50% LRRK2 kinase inhibition at 12 h after dosing. The plasma pharmacokinetic profile of PFE-360 can be found elsewhere (Andersen et al., 2018b).

### Motor symmetry assessment in the cylinder test

Nine weeks after the viral injection, motor function was assessed in the cylinder test. Rats receiving PFE-360 or vehicle were tested 1–2 h after dosing. Briefly, the rat was placed in a transparent cylinder and video recorded for 5 min. A minimum of 15 touches was required to ensure the sensitivity of the test. The ratio between the contralateral forepaw (to the injection) and the total number of touches was used as the primary read out.

### Spontaneous locomotor activity

Following the cylinder test, the non-forced spontaneous locomotor activity was evaluated in a cage similar to the home cage with clean bedding but without enrichment for 1 h. Activity was recorded by four photosensors along the cage. There was no habituation period. The activity count was calculated as the number of beam breaks, excluding repeated break of the same beam. Rats receiving PFE-360 or vehicle were tested 5–7 h after dosing.

### Single unit recordings of glutamatergic subthalamic neurons

Ten to 12 weeks following the viral vector injection, the same animals were subjected to single unit recording of putative glutamatergic STN neurons under urethane anesthesia (1.6–1.9 g/kg, i.p.). The level of anesthesia was maintained and monitored as absence of the deep pain reflexes but presence of the corneal reflex. This allowed to ensure that recordings were performed under similar levels of anesthesia, as the relative brain state influences the basal ganglia firing properties (Magill et al., 2000). The rat was placed in a stereotaxic frame and the body temperature was maintained at 37.5 ± 0.5°C. After a midline incision, a hole was drilled in os paritalis above the STN ipsilateral to the viral injection, at the following coordinates: 3.0–4.2 mm caudal to bregma and 2.4–2.8 mm lateral to the midline (Paxinos and Watson, 2007). The brain surface was kept moist with 0.9% saline after removal of the dura mater. The recording electrodes were fabricated from borosilicate glass capillaries (1B150F-4, World Precision Instruments) pulled into a fine tip under heating and broken under a microscope to a tip diameter of 3–8 μm reaching an *in vitro* resistance of 3–9 MΩ. The electrode was filled with 0.5 M sodium acetate containing 2% of Pontamine Sky Blue. The electrode was slowly lowered into STN using a motorized micromanipulator. STN was typically localized at 6.6–7.8 mm ventral to the brain surface (Paxinos and Watson, 2007). The action potentials were amplified (×10,000), band pass filtered (300 Hz to 5 kHz), discriminated and monitored on an oscilloscope and an audio monitor. Spike trains of action potentials were captured and analyzed using Spike 2 v 7.13 software with a computer-based system connected to the CED 1401 (Cambridge Electronic Design Ltd.). A minimum of 500 consecutive spikes was used for analysis of basal firing properties. PFE-360 was administrated to the rats per oral 1 h before the start of the recording session and ended 6–8 h after dosing.

At end of the recording session, an iontophoretic ejection of Pontamine Sky Blue was achieved by applying a negative voltage to the electrode allowing visualization of the last recording position using classical histologic methods. The recording sites were then retrospectively reconstructed, and neurons were only included in the analysis if the reconstruction provided evidence of the neuron to be within the STN.

### Single-unit recordings data analysis

The action potential of glutamatergic neurons in STN is typically short (<2 ms) with a biphasic wave form (Hollerman and Grace, 1992). Each spike-train was carefully inspected before the analysis using the principle component tools and the overdraw wavemark function in Spike 2 v 7.13, making sure that only spikes from a single neuron were included in the analysis. The signal-to-noise ratio was always >2:1. Spike trains were analyzed offline using a custom-made script measuring the firing rate and coefficient of variation of the interspike interval (CV ISI). Briefly, the CV ISI is defined as the ratio between the standard deviation of the ISI and the average ISI × 100 (Herrik et al., 2010). Further, the classification of the neuronal firing pattern was divided into regular, irregular, and bursty based on a visual inspection of the autocorrelogram and the discharge density histogram (Tepper et al., 1995; Kaneoke and Vitek, 1996).

### *Ex vivo* biochemical methods

#### Brain sampling

At the end of the recording session, the rats were perfused transcardially with 100-ml 0.9% saline containing 0.3% heparin. The brain was removed and sectioned on an ice-cold plate. The striata were snap frozen on dry-ice and stored at –80°C until preparation of tissue lysates for SDS-PAGE and Western blotting. The caudal part of the brain containing STN was stored at –20°C for histologic validation of the recording sites. The cerebellum was weighted and snap frozen in a Covaris tube on dry ice and stored at –80°C until further analysis.

### SDS-PAGE and Western blotting sample preparation

Preparation of striatal samples for SDS-PAGE and Western blotting was performed as described previously (Andersen et al., 2018a).

### SDS-PAGE and Western blotting quantification of tyrosine hydroxylase, striatal enriched phosphatase, hwt α-synuclein, and phosphorylated α-synuclein-S129

Quantification of TH, STEP and hwt-α-synuclein expression and α-synuclein-S129 phosphorylation were done as described previously (Andersen et al., 2018a; TH: Millipore catalog #AB152, RRID:AB_390204, STEP: LifeSpan catalog #LS-C2901, RRID:AB_2173553, hwt-α-synuclein: Abcam catalog #ab1904, RRID:AB_302666 and α-synuclein-S129 phosphorylation: Abcam catalog #ab51253, RRID:AB_869973). Briefly, the quantification was done using SDS-PAGE and subsequent Western blotting. Finally, the proteins were visualized quantified by infrared detection using Li-Cor Odyssey CLx (Li-COR).

### Total LRRK2 and phosphorylated LRRK2-S935 quantification

Total LRRK2 expression levels and the level of LRRK2 inhibition were quantified using SDS-PAGE and subsequent Western blotting as described previously (Andersen et al., 2018b; LRRK2: UC Davis/NIH NeuroMab Facility catalog #73-253, RRID:AB_10671178 and phosphorylated LRRK2-S935: Abcam catalog #ab133450, RRID:AB_2732035). The total LRRK2 was quantified for both ipsilateral and contralateral striatum and reported as the mean of the two measurements.

### Quantification of PFE-360 brain exposure

The analysis method is described previously (Andersen et al., 2018a). Briefly, the quantification of PFE-360 brain exposure was performed using a LC-MS/MS coupled to a Waters Acquity UPLC.

### Statistical analysis

Neuronal firing properties (firing rate and CV ISI) data were analyzed using Kruskal–Wallis test with Dunn’s corrected *p* value for multiple comparison, as they were non-normally distributed. Distributive differences in firing pattern were statistically assessed using χ^2^ analysis. The *p* value for multiple χ^2^ tests were corrected using the Bonferroni correction (*p* < 0.0125 was considered significant). Motor behavior and Western blotting quantifications (TH, STEP, α-synuclein-pS129, LRRK2, and LRRK2-pS935) data were all normally distributed and were analyzed using a one-way ANOVA test with Tukey’s *post hoc* multiple comparison. Statistical analysis of human α-synuclein overexpression and unbound PFE-360 exposure were done using an unpaired *t* test. All statistics and figures were made in GraphPad Prism 7.03. All values are presented as mean ± SEM.

## Results

### Chronic PFE-360 dosing modulates AAV-α-synuclein-induced motor deficit

**Figure 1.**
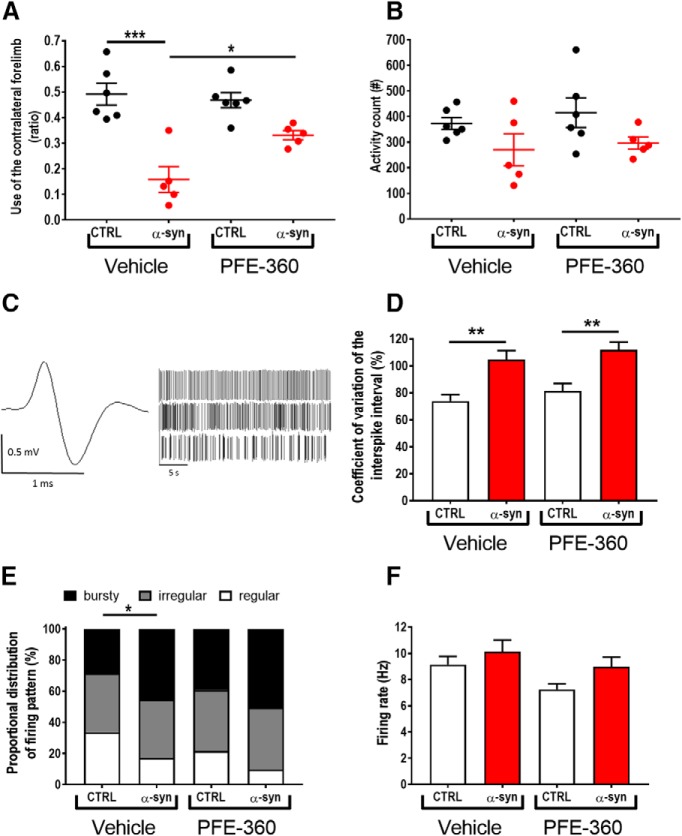
Chronic PFE-360 treatment slightly normalizes motor function but not STN burst firing induced by AAV-α-synuclein overexpression. ***A***, Motor symmetry assessed nine weeks after intracerebral viral inoculation of AAV-α-synuclein or CTRL (empty AAV-vector). The test was performed 1–2 h after dosing (one-way ANOVA, *p* < 0.001; CTRL veh and PFE-360 *N* = 6, α-syn veh and PFE-360 *N* = 5). ***B***, Locomotor activity measured 5–7 h after dosing revealed a non-significant trend for a decreased activity in α-synuclein overexpressing rats in both treatment groups compared to CTRL rats. PFE-360 treatment did not have any adverse effect on locomotion in the CTRL group (one-way ANOVA, *p* = 0.51; CTRL veh and PFE-360, *N* = 6, α-syn veh and PFE-360, *N* = 5). ***C***, Representative action potential and spike-trains from single unit recordings of putative glutamatergic neuron in the STN. The spike-trains are representative examples of regular (top), irregular (middle), and bursty (bottom) firing patterns. ***D***, CV ISI is increased by α-synuclein overexpression in both treatment groups (Kruskal–Wallis test, *p* < 0.001). ***E***, Proportional distribution of regular, irregular and bursty neurons (χ^2^ = 22.28, df = 6, *p* = 0.0011) shows increased proportion of bursty neurons in α-synuclein overexpressing rats in both treatment groups. ***F***, The average firing rate of STN neurons is not significantly different between groups (Kruskal–Wallis test, *p* = 0.11). ***D–F***, *N*: number of animals; *n*: number of neurons; CTRL veh (*N* = 5, *n* = 98), α-synuclein veh (*N* = 6, *n* = 99), CTRL PFE-360 (*N* = 6, *n* = 90), and α-synuclein PFE-360 (*N* = 6, *n* = 113). Tukey’s multiple comparison is represented by the lines; *p < 0.05, **p < 0.01, ***p < 0.001. For multiple χ^2^ tests in (***E***) the Bonferroni-corrected *p* value was used, *p < 0.0125 (four comparisons: significance level, *p* = 0.0125).

In our effort to evaluate the therapeutic preclinical potential of chronic LRRK2 inhibition, we first tested the effect of PFE-360 (7.5 mg/kg BID) on motor symmetry in the cylinder test. Overall, there was a statistically significant effect on the forepaws ratio across groups (Fig. 1*A*). Multiple comparisons showed that AAV-overexpression of α-synuclein induced a significant asymmetry in the motor function compared to an empty vector in vehicle-treated animals. Chronic dosing with PFE-360 did not affect the motor symmetry assessed in the cylinder test in animals injected with an empty vector. In rats injected with AAV-α-synuclein, PFE-360 partially normalized the dysfunctional use of forepaws as evidenced by the significantly increased use of the contralateral forepaw compared to vehicle treatment.

Analysis of the spontaneous locomotor activity 5–7 h after the last dosing did not show any overall statistical effect of either the viral vector or the treatment, although a trend for reduced locomotor activity was observed in animals injected with AAV-α-synuclein compared to an empty vector (Fig. 1*B*).

### AAV-α-synuclein-induced aberrant STN burst firing is not attenuated by chronic LRRK2 inhibition using PFE-360

To investigate the modulatory effect of repeated LRRK2 inhibition on STN burst firing induced by hwt α-synuclein overexpression in substantia nigra, rats were repeatedly dosed with PFE-360 for 10–12 weeks following viral injection. The electrophysiological properties of putative glutamatergic neurons in STN were recorded at the end of the treatment period, and 1–8 h following the last dose. A total of 400 putative glutamatergic STN neurons from 22 rats divided into four groups were included in the final analysis (Fig. 1*C–F*). Overall, comparison of the STN firing pattern revealed a significant group effect in the distribution of regular, irregular and bursty firing neurons between groups and a significant group effect in the CV ISI (Fig. 1*D*,*E*). Multiple comparisons revealed that overexpression of α-synuclein significantly increased the proportion of burst firing neurons and the CV ISI compared to an empty vector in vehicle-treated animals. In empty vector-injected animals, chronic dosing with PFE-360 7.5 mg/kg BID did not alter the relative distribution of the firing pattern or the CV ISI (CTRL vehicle vs CTRL PFE-360 7.5 mg/kg). In AAV-α-synuclein injected animals, chronic dosing with PFE-360 did not have any significant effect on the relative distribution of the firing pattern or the CV ISI. The CV ISI was significantly increased compared to CTRL PFE-360, to a level comparable to α-syn PFE-360. The relative distribution of firing pattern of α-synuclein was not significantly altered compared to CTRL PFE-360 or α-synuclein vehicle groups. The firing rate was not overall significant different between groups (Fig. 1*F*).

### Striatal LRRK2 expression is not decreased following chronic LRRK2 inhibition

**Figure 2.**
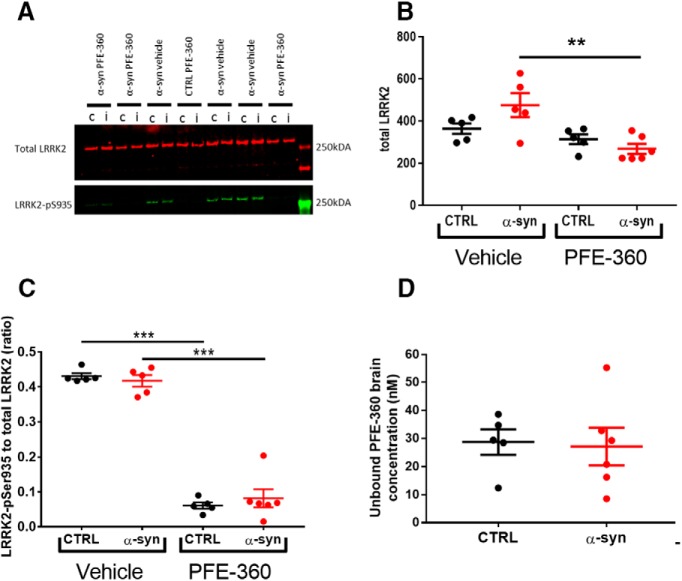
Striatal LRRK2 expression is not changed by PFE-360 treatment despite full LRRK2 inhibition. ***A***, Western blot analysis of total LRRK2 and phosphorylated LRRK2-Ser935 (LRRK2-pSer935) after 10- to 12-week chronic PFE-360 dosing. The tissues were collected 6–8 h after last dosing. i = ipsilateral to the viral injection; c = contralateral to the viral injection. ***B***, Quantification of Western blottings shows that chronic PFE-360 treatment did not affect LRRK2 expression in CTRL rats compared to vehicle treatment. In α-syn rats, PFE-360 treatment significantly lowered LRRK2 expression compared to vehicle treatment, although each α-syn group did not differ significantly from their respective CTRL group (one-way ANOVA, *p* = 0.0032). ***C***, The LRRK2-pS935 to total LRRK2 ratio was used as a measure of target engagement. At full inhibition, the ratio is typically <0.1 and was achieved in both CTRL and α-syn rats treated with PFE-360 (one-way ANOVA, *p* < 0.001). ***D***, Unbound PFE-360 brain concentrations 6–8 h after dosing. IC_50_ of PFE-360 was previously calculated to 2.3 nM giving theoretical 99% LRRK2 inhibition at 23 nM (unpaired *t* test, *p* = 0.85). CTRL veh, CTRL PFE-360 and α-syn veh; *N* = 5, α-syn PFE-360 *N* = 6. Tukey’s multiple comparison is represented by the lines; **p < 0.01, ***p < 0.001.

Evaluation of the impact of PFE-360 on total LRRK2 expression levels in striatum revealed an overall significant difference between groups (Fig. 2*A*,*B*). *Post hoc* analysis revealed no significant effect of AAV-α-synuclein overexpression in vehicle treated rats, nor was there any significant effect of PFE-360 treatment in CTRL rats. However, PFE-360 treatment in AAV-α-synuclein overexpressing rats significantly decreased the LRRK2 expression level compared to vehicle treated α-synuclein overexpressing rats. There was no statistical difference between PFE-360-treated CTRL and α-synuclein rats.

### PFE-360 exposure profile and target occupancy in rat brain

LRRK2 engagement and kinase inhibition in striatum of PFE-360 6–8 h after dosing was evaluated using SDS-PAGE and Western blotting (Dzamko et al., 2010, 2012; Nichols et al., 2010). Viral α-synuclein overexpression did not impact levels of LRRK2-pSer935 or total LRRK2 ratio (Fig. 2*C*). In CTRL and α-synuclein rats dosed with PFE-360 significant decreases in LRRK2-pSer935 to total LRRK2 ratio were observed. The result suggests that extended dosing of PFE-360 at 7.5 mg/kg for 10–12 weeks gives full LRRK2 kinase inhibition 6–8 h after dosing. A single rat exhibiting a PFE-360 exposure level ∼3 × IC_50_, suggestive of 70% inhibition at 8 h after dosing, was nevertheless included in the study (Fig. 2*D*). The calculated LRRK2 IC_50_ value and inhibition level is based on a single dose study using 7.5 mg/kg PFE-360 (Andersen et al., 2018a). Based on the exposure levels following a single PFE-360 dose of 7.5 mg/kg, the average level of LRRK2 inhibition in the current study is estimated to be from 100% to 50% over 24 h, fluctuating between 100% at 1–10 h after dosing and 100–50% at 10–12 h after dosing (data shown previously; Andersen et al., 2018a).

### LRRK2 inhibition does not attenuate the loss of TH expression in striatum induced by α-synuclein overexpression

**Figure 3.**
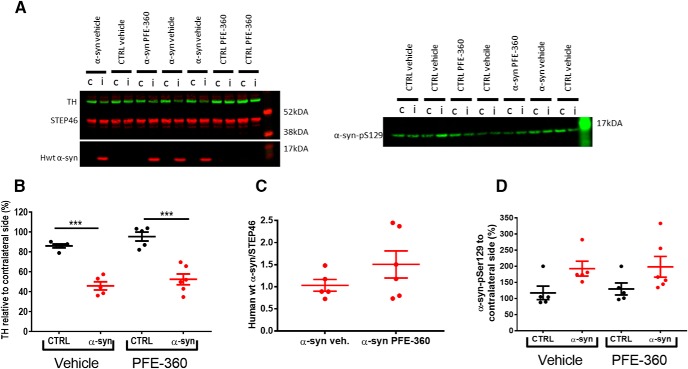
Striatal neurodegenerative processes and α-synuclein phosphorylation are not halted by chronic LRRK2 inhibition. ***A***, Western blot analysis of tyrosine hydroxylase (TH) and hwt α-synuclein (hwt α-syn; left) as well as phosphorylation of α-synuclein-S129 (α-syn pS129; right). i = ipsilateral to the viral injection; c = contralateral to the viral injection. ***B***, Quantification of TH expression from ipsilateral and contralateral striatum normalized to STEP46 and presented as percentage of contralateral striatum shows a significant loss of TH expression in α-syn overexpressing rats in both treatment groups (one-way ANOVA, *p* < 0.001). ***C***, The expression level of hwt α-synuclein normalized to STEP46 is not significantly different between groups (unpaired *t* test, *p* = 0.22). ***D***, The α-synuclein-S129 phosphorylation from ipsilateral and contralateral striatum normalized to STEP46 and presented as percentage of contralateral striatum is not significantly different between groups (one-way ANOVA, *p* = 0.076). CTRL veh, CTRL PFE-360 and α-syn veh; *N* = 5, α-syn PFE-360 *N* = 6. Tukey’s multiple comparison is represented by the lines; ***p < 0.001.

Neurodegenerative processes induced by AAV-α-synuclein overexpression were assessed indirectly by quantifying the expression level of TH in striatum. Overall, there was a significant effect of groups on striatal TH expression levels (Fig. 3*A*,*B*). *Post hoc* analysis revealed a significant loss of striatal TH induced by AAV-α-synuclein overexpression compared to an empty vector independently of treatment.

### LRRK2 inhibition does not affect human wt α-synuclein expression or phosphorylation of total α-synuclein

The overexpression of hwt α-synuclein was not significantly different between PFE-360 and vehicle-treated animals (Fig. 3*A*,*C*). The levels of pS129-α-synuclein in the striatum were overall not significantly different across groups (Fig. 3*A*,*D*). A trend for increased expression was observed following AAV-α-synuclein overexpression compared to CTRL animals although this was not significant. Chronic LRRK2 inhibition with PFE-360 did not impact α-synuclein-pS129 levels compared to vehicle in CTRL or AAV-α-synuclein rats.

## Discussion

Firstly, the findings suggest that the use of our current preclinical PD model in terms of predicting LRRK2 inhibitor efficacy in the clinic should be highly cautioned. Secondly, further investigation into mechanisms underlying these differences between models and investigators is needed before any solid conclusions can be made on the translational value of our current preclinical PD model. Finally, the discrepancy between the reported acute and present chronic effects of LRRK2 inhibition on aberrant α-synuclein-induced STN firing activity represents an interesting aspect of LRRK2 as a regulator of basal ganglia neurotransmission. Previous reports using acute or chronic LRRK2 inhibition have shown preclinical modulatory and protective effects of LRRK2 inhibition in α-synuclein-based PD models, supporting a pathophysiological interaction between LRRK2 kinase function and α-synuclein-induced pathology (Lin et al., 2009; Daher et al., 2015; Andersen et al., 2018a). In the present report, chronic LRRK2 inhibition had a marginal restorative effect on the motor performance evaluated in the cylinder test. The apparent PFE-360-induced improvement in motor function was not correlated to a similar reversal of STN burst firing. It should however be noted that the evaluation of forepaw use typically requires larger group size than used in the present study. The main aim of the cylinder test was to evaluate the efficiency of α-synuclein overexpression, which was indeed achieved as indicated by the strong motor asymmetry. Unfortunately, the small group size did not allow to make a definite conclusion on the effect of PFE-360 due to the minor level of significance of the reversal. In the event of an actual effect of PFE-360 on motor function, this would suggest that chronic LRRK2 inhibition may exert effects on motor function through action on other pathways bypassing STN, e.g., affecting neurotransmission and/or synaptic plasticity within the motor circuit, thereby functionally circumventing the relative strength/importance of STN firing on the motor output. The complexity of the motor circuit, where both strength and timing of a signal through the four pathways in the basal ganglia are important in determining the motor outcome (Nambu et al., 2002; Mastro and Gittis, 2015), may contribute to the possible discrepancy between the effects of PFE-360 on motor behavior and STN activity. Changes in synaptic plasticity have been reported in transgenic animals overexpressing LRRK2 (Beccano-Kelly et al., 2015; Sweet et al., 2015), but no data from LRRK2 knock-out animals is available. In the latter, it is conceivable that LRRK2 ablation from conception may induce compensatory mechanisms which would not be observed in wild-type animals unless long-term pharmacological inhibition would trigger similar compensations. Another possibility is that the function of LRRK2 inhibition needs an external trigger to reveal its functional involvement in neurotransmission such as impaired DAergic neurotransmission in substantia nigra and striatum. In this respect, alterations in glutamatergic transmission in striatal slices from G2019S-LRRK2 transgenic animals were only observed when rotenone, a mitochondrial toxin, was also present, indicating that LRRK2 might only play a role in the diseased state, such as impaired DAergic neurotransmission (Tozzi et al., 2018). In support, recent studies have suggested that LRRK2 kinase activity is increased in substantia nigra in the AAV-α-synuclein overexpression model and in rotenone models as well as in postmortem tissue from idiopathic PD patients (Howlett et al., 2017; Di Maio et al., 2018).

The acute LRRK2 inhibition with PFE-360 was shown to drastically reduce the spontaneous locomotor activity of control animals (Andersen et al., 2018a), a hypolocomotor effect was not observed after repeated PFE-360 treatment in the present study. This potentially indicates an adaptation to the hypolocomotor effect after repeated administration. Our target engagement measurements showed full LRRK2 inhibition in the brain after repeated treatment (and periphery; data not shown; Andersen et al., 2018b), but no consistent change in LRRK2 expression, thereby ruling out adaptive effects mediated directly by LRRK2 and further suggesting the existence of adaptive mechanisms taking place downstream of LRRK2. Further investigation in long-term treated subjects preclinically and clinically will be needed to validate such physiologic effects.

Our investigation recapitulated the effect of AAV-α-synuclein overexpression on STN basal firing properties by increasing the proportion of burst firing neurons, mimicking findings in neurotoxin models and PD patients (Hollerman and Grace, 1992; Bergman et al., 1994; Ni et al., 2000; Iancu et al., 2005; Brazhnik et al., 2014; Pan et al., 2016). As above mentioned, chronic LRRK2 inhibition using PFE-360 was unable to counteract AAV-α-synuclein-induced aberrant STN burst firing. Previously, aberrant STN firing in the AAV-α-synuclein rat model is reported to be normalized by acute LRRK2 inhibition using PFE-360 (Andersen et al., 2018a). In the present study, all putative STN neurons were recorded under 70–100% LRRK2 inhibition (based on theoretical back calculations of brain exposure over time; data not shown). The discrepancy between the effect of acute and chronic LRRK2 inhibition on STN firing properties further supports induction of downstream adaptation to PFE-360 after long-term exposure, which was also observed in the locomotor test. Recent investigation has functionally linked LRRK2 activation of RAB35 GTPase to the propagation of α-synuclein pathology, together with a number of other RAB GTPases (e.g., Rab3A/B/C/D, Rab8A/B, Rab10, Rab12, Rab29, Rab35, and Rab43; Beilina et al., 2014; Steger et al., 2016; Alessi and Sammler, 2018; Bae et al., 2018). The functional diversity of the RAB GTPase family highly complicates the relationship between LRRK2 inhibition, STN firing pattern and motor function. The involvement of RAB GTPases in cellular trafficking puts the regulatory potential of LRRK2 on neurotransmission in a vulnerable position, in modulating both pathogenesis and synaptic function. A shift in the membrane-cytosol RAB GTPase homeostasis caused by chronic LRRK2 inhibition has the potential to alter intracellular trafficking (Steger et al., 2016). This in turn may indirectly modulate neurotransmission both presynaptically and postsynaptically, e.g., by interfering with neurotransmitter release and/or reuptake and changing ion-channel membrane density, as described in other tissues as well as in neurons (Farinha and Matos, 2018; Mignogna and D’Adamo, 2018). In this perspective, reversal of the PD-like motor phenotype might take place at any place in motor related nuclei downstream of STN. Substantia nigra pars retigulata receives glutamatergic input from STN and is the main output from the basal ganglia sending projections to the mesencephalic locomotor region (MLR) in the midbrain. Direct unilateral activation of MLR can evoke bilateral locomotor behavior (Caggiano et al., 2018), a regulation of synaptic function at this level have the potential modulate motor output.

We further showed that chronic treatment with PFE-360 did not have any impact on DAergic neurodegenerative processes, striatal hwt-α-synuclein expression or striatal α-synuclein-pS129 level induced by AAV-α-synuclein overexpression in substantia nigra and associated pathways. The level of Striatal TH expression is previously shown to correlate well with the level of neurodegeneration in SNc (Oliveras-Salvá et al., 2013). The findings contrast findings by Daher et al. (2015) who reported protection of DAergic neurons from α-synuclein-induced neurodegeneration after chronic treatment with a similar LRRK2 inhibitor chemotype (Daher et al., 2015). A possible explanation for this discrepancy is related to the different design of the respective studies. In the study by Daher et al. (2015), animals were assessed four weeks following viral injection and drug treatment initiation, which may have allowed revealing a delay in the progression of the pathology. The viral serotype used was also different in their study compared to the present, which can have important impact on the infectious potential of the viral vector (Van der Perren et al., 2011). Importantly, the treatment regimen used in our study did not result in full LRRK2 inhibition throughout the whole duration of the treatment based on the free brain concentration of PFE-360 at 12 h after dose (data from acute administration of PFE-360; Andersen et al., 2018a). Whether full brain LRRK2 inhibition was achieved throughout the study reported by Daher et al. (2015) is unknown. Thus, to fully explore the potential of LRRK2 kinase inhibitors in preclinical models, further development of novel LRRK2 inhibitors with more optimal pharmacokinetic and pharmacodynamic properties is needed or a study design reaching full LRRK2 inhibition 24 h/d. An alternative explanation for the lack of LRRK2 kinase inhibitor effects could relate to the expression pattern of LRRK2 in rats. A recent study has shown that the LRRK2 expression is undetectable in DAergic neurons (West et al., 2014), excluding direct interaction between LRRK2 and α-synuclein in our study. Endogenous LRRK2 in rats is mainly localized to cortico-striatal neurons and MSNs (Davies et al., 2013; West et al., 2014).

In the literature it has consistently been observed that other kinases than LRRK2 phospshorylate the Ser935 site but only if ATP is bound in the catalytic domain and LRRK2 attains an active conformation (Dzamko et al., 2010, 2012; Nichols et al., 2010). Thus, we used the phosphorylation of Ser935-LRRK2 as a surrogate marker of target engagement and full target engagement of LRRK2 is also equivalent with full LRRK2 inhibition and loss of LRRK2 enzymatic activity. It is further shown that the IC50 value for LRRK2 inhibition with PFE-360 is the same for pThr73-Rab10 as the IC50 for LRRK2 inhibition on pSer935-LRRK2 (Thirstrup et al., 2017).

Chronic treatment with PFE-360 in CTRL rats was not associated with a change in LRRK2 expression in the striatum *in vivo*. Although LRRK2 expression was significantly lower in rats overexpressing α-synuclein treated with PFE-360 compared to vehicle, α-synuclein overexpression in vehicle-treated rats was not associated to any significant change in LRRK2 expression compared to Ctrl rats. The apparent difference in LRRK2 expression between PFE-360- and vehicle-treated α-synuclein overexpressing rats may therefore result from methodological variation rather than a pathophysiological effect. Previous studies in the AAV-α-synuclein rat model have not found similar effects of α-synuclein overexpression on LRRK2 expression (Andersen et al., 2018a). The lack of changes in LRRK2 expression after chronic LRRK2 inhibition in CTRL rats is in agreement with similar findings in the brain after 11 d of chronic treatment with MLi-2 (Fell et al., 2015). In contrast, others have reported large decreases in total brain LRRK2 expression *in vivo* and *in vitro* in cellular systems after LRRK2 kinase inhibition (Herzig et al., 2011; Daher et al., 2015; Fuji et al., 2015; Zhao et al., 2015; Lobbestael et al., 2016). In this regard, the species (rat vs mouse vs non-human primate) and LRRK2 expression level should be considered. *In vitro* overexpression of LRRK2 might be very different from *in vivo* conditions with much lower expression of LRRK2 *in vivo* and might represent different mechanisms not allowing direct comparison.

Although the viral overexpression model has considerably increased our understanding of α-synuclein-induced alterations in the basal ganglia circuitry, the α-synuclein preformed fibrils model has been recently suggested to represent a more faithful model of idiopathic PD (Duffy et al., 2018), and may therefore be highly valuable to further validate LRRK2 as a therapeutic target for idiopathic PD.

## Summary

We have demonstrated that chronic LRRK2 inhibition induced a marginal reversal of the motor dysfunction in the AAV-α-synuclein rat model of PD. This effect was not associated with any beneficial effect on STN burst firing or loss of striatal TH expression. The discrepancies between previously reported acute effects and the present chronic effects of LRRK2 inhibition may be dependent on compensatory mechanisms after chronic LRRK2 inhibition, or on the limited validity of the AAV overexpression model as a PD model. Further studies in other preclinical models such as the α-synuclein PFF model may prove valuable.

In the search for the precise mechanisms underlying a putative interaction between LRRK2 and α-synuclein, changes in synaptic plasticity and vesicular trafficking seems to play a pivotal role in model systems manipulating neuronal and non-neuronal LRRK2 kinase function, respectively. Such changes might result in wanted or unwanted effects. It was previously described that LRRK2 is involved in synaptic plasticity and that it is dysregulated in the parkinsonian state (Beccano-Kelly et al., 2015; Chu et al., 2015; Mastro and Gittis, 2015; Sweet et al., 2015), thereby strengthening a potential beneficial effect of LRRK2 inhibition on synaptic plasticity in the pathologic conditions.

## Synthesis

Reviewing Editor: Dritan Agalliu, Columbia University Medical Center Decisions are customarily a result of the Reviewing Editor and the peer reviewers coming together and discussing their recommendations until a consensus is reached. When revisions are invited, a fact-based synthesis statement explaining their decision and outlining what is needed to prepare a revision will be listed below. The following reviewer(s) agreed to reveal their identity: Anders Borgkvist.

This manuscript provides only a small advance in the field. Using an established model of Parkinson's disease (overexpression of alpha-synuclein), the authors find that an LRRK2 inhibitor rescue Parkinsonism in the cylinder test. However, they do not examine the mechanisms for this therapeutic effect. In contrast, the authors find that pathogenic markers, specifically the beta-rhythm in the subthalamic nucleus that has been shown in various animal models to correlate with parkinsonisms, is unchanged by the pharmacological treatment. Therefore, drawing any conclusions about this specific finding is difficult.

Reviewer # 1:

Major Concerns:

1) The most critical finding of the paper is that the LRRK2 inhibitor increases forepaw use in the cylinder test. However, this test is not particularly conclusive. The authors should perform a more comprehensive behavioral analysis before concluding therapeutic efficacy. For example, they should include rotational behavior test with amphetamine and the rotarod test. I am also surprised about the strong forepaw bias the authors find in the cylinder test. Typically a 10-15% decrease in paw use with 80% dopamine lesions can be observed, while in this paper the authors see a reduction in paw use by more than 30% with only 40% reduction in TH neurons. This discrepancy with the literature validates a more rigorous behavioral testing.

2) In the cylinder test, the authors should test a separate LRRK2 inhibitor in parallel to see if the observed effect is consistent.

3) In the western blot analysis, the authors should load triplicates of the control group (vehicle-ctrl rats) in each gel they perform and express the ratio of change according to the average of these triplicates. Otherwise, analyses are invalid. In Fig 2A, I see no control group at all. Please clarify how the total LRRK2 values were obtained if no control groups were run on the same gel.

4) In the gel in Fig 2, it looks like the unilateral alfa-synuclein overexpression leads to more LRRK2 expressed bilaterally. How do the authors explain these findings? How does this affect the interpretation of the one-sided bias in the behavior? Please clarify these inconsistencies.

4) In the absence of stereological counts of the dopamine denervation, the authors should also include a western blot showing DAT expression.

Minor Concerns:

1) Please specify which AAV vector was used in the study.

Reviewer # 2:

Major Concerns:

1) Experiments using AAV-alpha-synuclein lack proper controls. The control AAV should express a protein, not just an empty capsid.

2) In Figure 1A-B, the authors demonstrate a partial rescue of forelimb asymmetry with the administration of PFE-360. Authors should support this finding through the use of other behavioral tests for disease-associated motor phenotypes. In addition, they should measure striatal dopamine.

3) In Figure 1C-F, the authors perform electrophysiological recordings from cells in the substantia nigra to investigate the effect of PFE-360 on the disease model. The authors should confirm that the neurons being recorded from express LRRK2 in order to draw conclusions about the effect of the drug. There is also no data or discussion about which cell types in the brain express LRRK2 and whether the drug has an effect in those cell types.

3) While authors provide evidence that PFE-360 inhibits LRRK2 autophosphorylation at serine 935 and that PFE-360 brain concentration is not affected by alpha-synuclein overexpression, this is an inadequate characterization of the drug. Pharmacokinetic studies are necessary in order to determine drug properties in the CNS. Additionally, LRRK2 can act on targets other than the autophosphorylation site on serine 935. PFE-360 effects on other substrates should be investigated and discussed.

4) In Figure 3A, authors demonstrate that PFE-360 does not alter TH expression. However, the effect of PFE-360 on neurodegeneration needs to be investigated using unbiased stereology of the substantia nigra.

## Author Response

Revised manuscript: Long-term exposure to PFE-360 in the AAV-α-synuclein rat model: Findings and inplications

Dear Dritan Agalliu

We are thankful for the constructive and helpful criticism that has been raised by yourself and the reviewers and would hereby like to submit a revised version of our manuscript.

Based on the different comments, the revised version includes changes and additions as listed in the remaining of this letter:

Synthesis of Reviews:

Significance Statement Comments for Author (Required):

The authors need to revise the Significance Statement to describe more in detail their study and the significance of their findings.

To address this comment, we have emphasised that the main finding from this study is the different outcome of chronic PFE-360 treatment on α-synuclein-induced phenotype compared to an acute treatment with the same inhibitor reported in a previous study.

Synthesis Statement for Author (Required):

This manuscript provides only a small advance in the field. Using an established model of Parkinson's disease (overexpression of alpha-synuclein), the authors find that an LRRK2 inhibitor rescue Parkinsonism in the cylinder test. However, they do not examine the mechanisms for this therapeutic effect. In contrast, the authors find that pathogenic markers, specifically the beta-rhythm in the subthalamic nucleus that has been shown in various animal models to correlate with parkinsonisms, is unchanged by the pharmacological treatment. Therefore, drawing any conclusions about this specific finding is difficult.

As we eluded to above, the main finding from the present studies is the overall lack of significant effect of chronic treatment with PFE-360 on α-synuclein-induced phenotype in a rat AAV model, which is in contrast with our previous report of a beneficial effect of an acute treatment with PFE-360 on STN aberrant firing in the same model. In addition, our results are in contrast with other findings where chronic LRRK2 inhibition was reported to protect against α-synuclein-induced neurodegeneration in a similar AAV rat model. We believe the findings from the present studies further outline some of the limitations inherent to the PD model used, and warrant additional preclinical studies in animal models with better relevance to the clinical pathophysiology, e.g. fibril-induced seeding.

Reviewer # 1:

Major Concerns:

1) The most critical finding of the paper is that the LRRK2 inhibitor increases forepaw use in the cylinder test. However, this test is not particularly conclusive. The authors should perform a more comprehensive behavioral analysis before concluding therapeutic efficacy. For example, they should include rotational behavior test with amphetamine and the rotarod test. I am also surprised about the strong forepaw bias the authors find in the cylinder test. Typically a 10-15% decrease in paw use with 80% dopamine lesions can be observed, while in this paper the authors see a reduction in paw use by more than 30% with only 40% reduction in TH neurons. This discrepancy with the literature validates a more rigorous behavioral testing.

The present findings in the cylinder test indicate the presence of a motor asymmetry induced by α-synuclein, which seems to be partially reversed by chronic treatment with PFE-360. However, it should be noted that the evaluation of forepaw use typically requires larger group size than used in the present study and differences in activity levels between groups might interfere with the sensitivity of the test. The main aim was to evaluate the efficiency of α-synuclein overexpression. We and others have previously reported a decreased ratio in this test prior to overt dopaminergic neurodegeneration (Andersen et al., 2018a) indicating that the α-synuclein load in DA neurons is a significant contributing factor in the observed motor impairment, possibly through impairing DA neurotransmission in the striatum, as indicated by changes in DA cell firing activity and DA homeostasis in the striatum (Gaugler et al., 2012; Lundblad et al., 2012; Janezic et al., 2013). Therefore, motor asymmetry can be dependent on loss of DA neurons, but also on impaired DA neurotransmission without DA neuron loss as observed following α-synuclein overexpression. Our findings do support the presence of a motor asymmetry induced by α-synuclein overexpression in the present study. However, the small group size does not all to draw definite conclusions on the effect of PFE-360. In this respect, we have revised the text of the manuscript to attenuate the importance of this observation.

2) In the cylinder test, the authors should test a separate LRRK2 inhibitor in parallel to see if the observed effect is consistent.

This point is addressed above.

3) In the western blot analysis, the authors should load triplicates of the control group (vehicle-ctrl rats) in each gel they perform and express the ratio of change according to the average of these triplicates. Otherwise, analyses are invalid. In Fig 2A, I see no control group at all. Please clarify how the total LRRK2 values were obtained if no control groups were run on the same gel.

We acknowledge the reviewers concern; however, we are of the conviction that the analyses are still valid and can be used to support the findings. Figure 2A is a representative example of a Western Blot with two different samples from 7 different rats (mix of groups). The intend of Figure 2 is to show that i) dosing of 7.5mg/kg PFE-360 BID leads to full LRRK2 inhibition in rat brain by assessing total LRRK2 levels and phosphorylated LRRK2 at Ser935, and ii) LRRK2 levels are not decreased by inhibition. It is correct that the total LRRK2 signal was not normalised to other proteins nor set relative to control rats; however, input to lanes was carefully assessed by prior protein determination.

4) In the gel in Fig 2, it looks like the unilateral alfa-synuclein overexpression leads to more LRRK2 expressed bilaterally. How do the authors explain these findings? How does this affect the interpretation of the one-sided bias in the behavior? Please clarify these inconsistencies.

The reason for presenting the total LRRK2 signal was merely done in order to balance the discussion on previous reports in the literature where reduced levels of LRRK2 have been observed after chronic LRRK2 inhibition. In our experience, we have never observed that in brain nor kidney samples using our preferred lysis buffers and Western blot protocol for LRRK2 quantification. We thank the reviewers for the input and do agree that the difference in total LRRK2 levels is significantly increased in vehicle α-synuclein rats when compared to PFE-360 α-synuclein rats. The difference in LRRK2 expression between vehicle Ctrl rats and vehicle α-synuclein rats is not significant and as mentioned in text, the difference in the total LRRK2 signal between vehicle- and PFE-360-treated α-synuclein rats is more likely to represent methodological variation. This has now been cautioned and implemented in the text line 344 “Although LRRK2 expression was significantly lower in rats overexpressing α-synuclein treated with PFE-360 compared to vehicle, α-synuclein overexpression in vehicle-treated rats was not associated to any significant change in LRRK2 expression compared to Ctrl rats. The apparent difference in LRRK2 expression between PFE-360- and vehicle-treated α-synuclein overexpressing rats may therefore result from methodological variation rather than a pathophysiological effect.”

4) In the absence of stereological counts of the dopamine denervation, the authors should also include a western blot showing DAT expression.

It is correct that stereological counting of dopaminergic neurons has not been assessed. However, the main objective of the manuscript was not to describe the AAV-α-synuclein overexpression model and its functional consequences as this was previously described in a previous publication from our laboratories. In our extensive characterization of the AAV-α-synuclein overexpression model, we have observed that the same viral construct as used in the present study typically induced a 50% loss of TH expression in the striatum on the injected side (assessed by Western blot as well as densitometry analysis from TH immunostainings) after 8 to 10 weeks, and a corresponding 20% decrease in the number of TH-positive neurons in the SN pars compacta as assessed by stereology (unpublished observations). Based on these observations, we believe that the quantification of TH expression in the striatum can be used as a surrogate measure for the relative number of dopaminergic terminals in the striatum. This has been done in Figure 3A-B showing that unilateral expression of a-synuclein leads to an approximate 50% reduction of TH suggesting loss of 50% of the dopaminergic terminals in the striatum. DAT expression was not investigated in the present studies, as we have previously observed that DAT/TH ratio was increased 8 weeks post AAV-α-synuclein overexpression, possibly indicating a compensatory upregulation of DAT expression in the remaining terminals. Alternatively, we observed that VMAT2 expression was decreased in parallel with TH expression in the AAV-α-synuclein overexpression model (unpublished observations).

Minor Concerns:

1) Please specify which AAV vector was used in the study.

Thanks for observing this obvious mistake, the subtype has been added in line 87.

Reviewer # 2:

Major Concerns:

1) Experiments using AAV-alpha-synuclein lack proper controls. The control AAV should express a protein, not just an empty capsid.

There is a lot of debate in the scientific community on the best control to use for AAV-overexpression in vivo. We and others have previously reported adverse effects of GFP overexpression, one of the most commonly overexpressed control protein, including toxic effects as shown by reduced TH expression in striatum and number of TH positive cells in SNc (Koprich et al., 2010; Sanchez-Guajardo et al., 2010; Andersen et al., 2018a). Identification of the most optimal control construct remains unresolved. While the use of an empty vector is a commonly used alternative (Decressac et al., 2012a, 2012b), an AAV expressing a scrambled sequence related to α-synuclein may represent a more attractive option. However, we do not feel this has implications for the conclusions related to the effects of LRRK2 inhibition.

2) In Figure 1A-B, the authors demonstrate a partial rescue of forelimb asymmetry with the administration of PFE-360. Authors should support this finding through the use of other behavioral tests for disease-associated motor phenotypes. In addition, they should measure striatal dopamine.

We kindly refer to the answers provider in comment 1 and 2 from reviewer 1. Furthermore, the level of TH has previously been shown to parallel the DA levels in striatum in the AAV-α-synuclein rat model (Decressac et al., 2012a; Lundblad et al., 2012)

3) In Figure 1C-F, the authors perform electrophysiological recordings from cells in the substantia nigra to investigate the effect of PFE-360 on the disease model. The authors should confirm that the neurons being recorded from express LRRK2 in order to draw conclusions about the effect of the drug. There is also no data or discussion about which cell types in the brain express LRRK2 and whether the drug has an effect in those cell types.

The present studies investigate the effect of LRRK2 inhibition on neuronal activity in the subthalamic nucleus, a key area in the basal ganglia pathways, but not in the substantia nigra. There is no report of LRRK2 expression in the STN, however strong expression is present in the striatal MSNs, which are located upstream of the STN in the indirect pathway. Therefore, it is possible that modulation by LRRK2 of MSNs excitability may have an impact on the circuitry encompassing the STN. As shown in fig 2, the treatment regimen used in the represent studies resulted in strong reduction in pSer935-LRRK2 in striatum. For more background information on LRRK2 expression in rodents, a section has been included line 333

4) While authors provide evidence that PFE-360 inhibits LRRK2 autophosphorylation at serine 935 and that PFE-360 brain concentration is not affected by alpha-synuclein overexpression, this is an inadequate characterization of the drug. Pharmacokinetic studies are necessary in order to determine drug properties in the CNS. Additionally, LRRK2 can act on targets other than the autophosphorylation site on serine 935. PFE-360 effects on other substrates should be investigated and discussed.

It is correct that pharmacokinetic studies are not being presented in this report. We direct the reviewers towards previous publications showing the PK profile of PFE-360 in the rat (Andersen et al., 2018b, 2018a).

The phosphorylation of Ser935 is not an autophosphorylation; it has consistently been shown in the literature that other kinases than LRRK2 phosphorylate this site but only if ATP is bound in the catalytic domain. Thus, pSer935 levels is a surrogate marker for LRRK2 target engagement and full target engagement of LRRK2 is also equivalent with full LRRK2 inhibition and the loss of LRRK2 enzymatic activity. It is correct that there are validated bona fide LRRK2 substrates such as the small Rab GTPases Rab1, Rab3, Rab5, Rab8, Rab10, Rab12, Rab29, Rab35 and Rab43. However, studies have shown that the IC50 value for LRRK2 inhibition with PFE360 is the same for Rab10- pThr73 as the IC50 for LRRK2 inhibition on LRRK2-pSer935 (Thirstrup et al., 2017). We have included that in the text (line 342) that phosphorylation of Ser935-LRRK2 is an accepted surrogate measure of LRRK2 kinase activity.

5) In Figure 3A, authors demonstrate that PFE-360 does not alter TH expression. However, the effect of PFE-360 on neurodegeneration needs to be investigated using unbiased stereology of the substantia nigra.

As already stated above (reviewer 1, point 4), we have previously observed that a 50% loss of TH in striatum was associated with a 20% loss of cell bodies in the SNc 8 to 10 weeks following AAV overexpression using the same construct. TH loss in striatum also seems to appear first prior to any detectable cell loss in the SNc (unpublished observations), also in line with the terminals being the primary target of degeneration and the loss of cell bodies in PD resulting of a “dying back” process (Dauer and Przedborski, 2003). Furthermore, it is unlikely that the lack of effect of LRRK2 inhibition on neurodegenerative processes in the striatum would be associated to a beneficial effect on neurodegenerative processes in the SN.

References:

Andersen MA, Christensen KV, Badolo L, Smith GP, Jeggo R, Jensen PH, Andersen KJ, Sotty F (2018a) Parkinson's disease-like burst firing activity in subthalamic nucleus induced by AAV-α-synuclein is normalized by LRRK2 modulation. Neurobiol Dis 116:13-27.

Andersen MA, Wegener KM, Larsen S, Badolo L, Smith GP, Jeggo R, Jensen PH, Sotty F, Christensen KV, Thougaard A (2018b) PFE-360-induced LRRK2 inhibition induces reversible, non-adverse renal changes in rats. Toxicology 395:15-22.

Decressac M, Mattsson B, Lundblad M, Weikop P, Björklund A (2012a) Progressive neurodegenerative and behavioural changes induced by AAV-mediated overexpression of α-synuclein in midbrain dopamine neurons. Neurobiol Dis 45:939-953.

Decressac, Mattsson B, Björklund A (2012b) Comparison of the behavioural and histological characteristics of the 6-OHDA and α-synuclein rat models of Parkinson's disease. Exp Neurol 235:306-315.

Gaugler MN, Genc O, Bobela W, Mohanna S, Ardah MT, El-Agnaf OM, Cantoni M, Bensadoun J-C, Schneggenburger R, Knott GW, Aebischer P, Schneider BL (2012) Nigrostriatal overabundance of α-synuclein leads to decreased vesicle density and deficits in dopamine release that correlate with reduced motor activity. Acta Neuropathol 123:653-669.

Janezic S et al. (2013) Deficits in dopaminergic transmission precede neuron loss and dysfunction in a new Parkinson model. Proc Natl Acad Sci U S A 110:E4016-25.

Koprich JB, Johnston TH, Reyes MG, Sun X, Brotchie JM (2010) Expression of human A53T alpha-synuclein in the rat substantia nigra using a novel AAV1/2 vector produces a rapidly evolving pathology with protein aggregation, dystrophic neurite architecture and nigrostriatal degeneration with potential to model the pat. Mol Neurodegener 5:43.

Lundblad M, Decressac M, Mattsson B, Björklund A (2012) Impaired neurotransmission caused by overexpression of α-synuclein in nigral dopamine neurons. Proc Natl Acad Sci U S A 109:3213-3219.

Sanchez-Guajardo V, Febbraro F, Kirik D, Romero-Ramos M (2010) Microglia Acquire Distinct Activation Profiles Depending on the Degree of α-Synuclein Neuropathology in a rAAV Based Model of Parkinson's Disease Cookson MR, ed. PLoS One 5:e8784.

Thirstrup K, Dächsel JC, Oppermann FS, Williamson DS, Smith GP, Fog K, Christensen K V. (2017) Selective LRRK2 kinase inhibition reduces phosphorylation of endogenous Rab10 and Rab12 in human peripheral mononuclear blood cells. Sci Rep 7:10300.
